# Evaluating the equity of access to community health service centers in different regions of China based on cyclability and walkability

**DOI:** 10.3389/fpubh.2025.1563750

**Published:** 2025-04-28

**Authors:** Zhenhua Xu, Jiaying Chang, Cong Han

**Affiliations:** ^1^School of Urban Construction, Beijing City University, Beijing, China; ^2^Shenzhen International Graduate School, Tsinghua University, Beijing, China; ^3^Beijing Hengchuang Institute of Additive Manufacturing Research Co., Ltd., Beijing, China; ^4^School of Architecture and Art, Hebei University of Architecture, Hebei, China; ^5^School of Architecture, University of Manchester, Manchester, United Kingdom

**Keywords:** community health service centers, cyclability, walkability, GIS analysis, equity

## Abstract

With China’s rapid urbanization and aging population, ensuring equitable access to essential facilities in Community Health Service Centers (CHSCs) is critical for delivering primary healthcare to older adults. These centers serve as the frontline for managing chronic diseases, promoting healthy aging, and reducing healthcare disparities among the older adults. This study investigates the equity of CHSCs across eastern, central, and western regions of China using indicators of cyclability and walkability, both of which are essential for older populations who often rely on active travel modes due to physical or economic limitations. A total of 110 nationally ranked CHSCs were selected for analysis. Geographic Information System (GIS) technology was used to assess cyclability, defined by a 1,000-meter residential area coverage, and walkability, represented by the average Walk Score. Results show that the cyclability scores for the eastern, central, and western regions were 0.71, 0.64, and 0.46, respectively, below the national standard of a 1,000-meter service radius, highlighting insufficient cycling access to primary care for older residents. Walkability scores were 0.351, 0.388, and 0.287, with lower values, particularly concerning aging populations with limited mobility. These findings reveal pronounced regional disparities and point to the need for spatial optimization of CHSCs, increased facility provision, and targeted resource allocation to western regions. By enhancing the active accessibility of community healthcare services, particularly for older adults, this research provides policy-relevant evidence to advance equity, promote healthy aging, and support sustainable public health planning in China.

## 1 Introduction

### 1.1 Background of the study

#### 1.1.1 Equity disparities in community health service centers

Community health service centers (CHSCs) play a critical role in the foundational healthcare system, providing essential services such as preventive care, basic medical treatment, health education, and chronic disease management (1, 2). The quality and coverage of these services are directly connected to protecting residents’ health rights and interests (3). As primary public health service providers, CHSCs play a crucial role in addressing the challenges of an aging population and the high prevalence of chronic diseases (4). The equity of CHSCs influences not only the realization of residents’ health rights but also the equitable allocation of medical resources (5, 6). Equity is primarily reflected in the spatial distribution of CHSCs and the equitable access to and quality of medical services across regions (7). However, disparities in economic development, urbanization, and transportation infrastructure across China have resulted in significant imbalances in the distribution and service capacity of CHSCs, particularly concerning cyclability and walkability (8). These inequities directly impact the fairness and availability of healthcare services.

In economically developed regions, where medical resource allocation and infrastructure are more advanced, CHSCs are densely distributed, extensive service coverage, and residents benefit from convenient access to high-quality essential medical services (9). Conversely, insufficient public investment in less economically developed central and western regions leads to limited CHSCs with dispersed layouts, hindering efforts to meet residents’ healthcare needs (10). Additionally, disparities in transportation infrastructure intensify service inequalities across regions. In large cities and regions with well-developed transportation systems, residents can readily access CHSCs through multiple modes of transport, benefiting from greater service accessibility (11). Conversely, in regions with underdeveloped transportation networks, particularly rural areas, residents often spend considerable time or walk long distances to reach CHSCs, significantly raising the cost and effort of accessing healthcare services (12).

Against this backdrop, cyclability and walkability have become critical indicators for evaluating the equity of CHSCs. These indicators reflect the appropriateness of CHSC distribution and substantially impact residents’ utilization of healthcare services. Regional disparities in healthcare equity hinder the efficient use of medical resources and exacerbate health outcome inequalities, thereby obstructing progress toward the “Healthy China 2030″ initiative. Therefore, evaluating the equity of CHSCs through cyclability and walkability across regions is essential. This approach can optimize resource allocation and improve both accessibility and the efficiency of primary healthcare services.

#### 1.1.2 Serious aging in China

**Figure 1:**
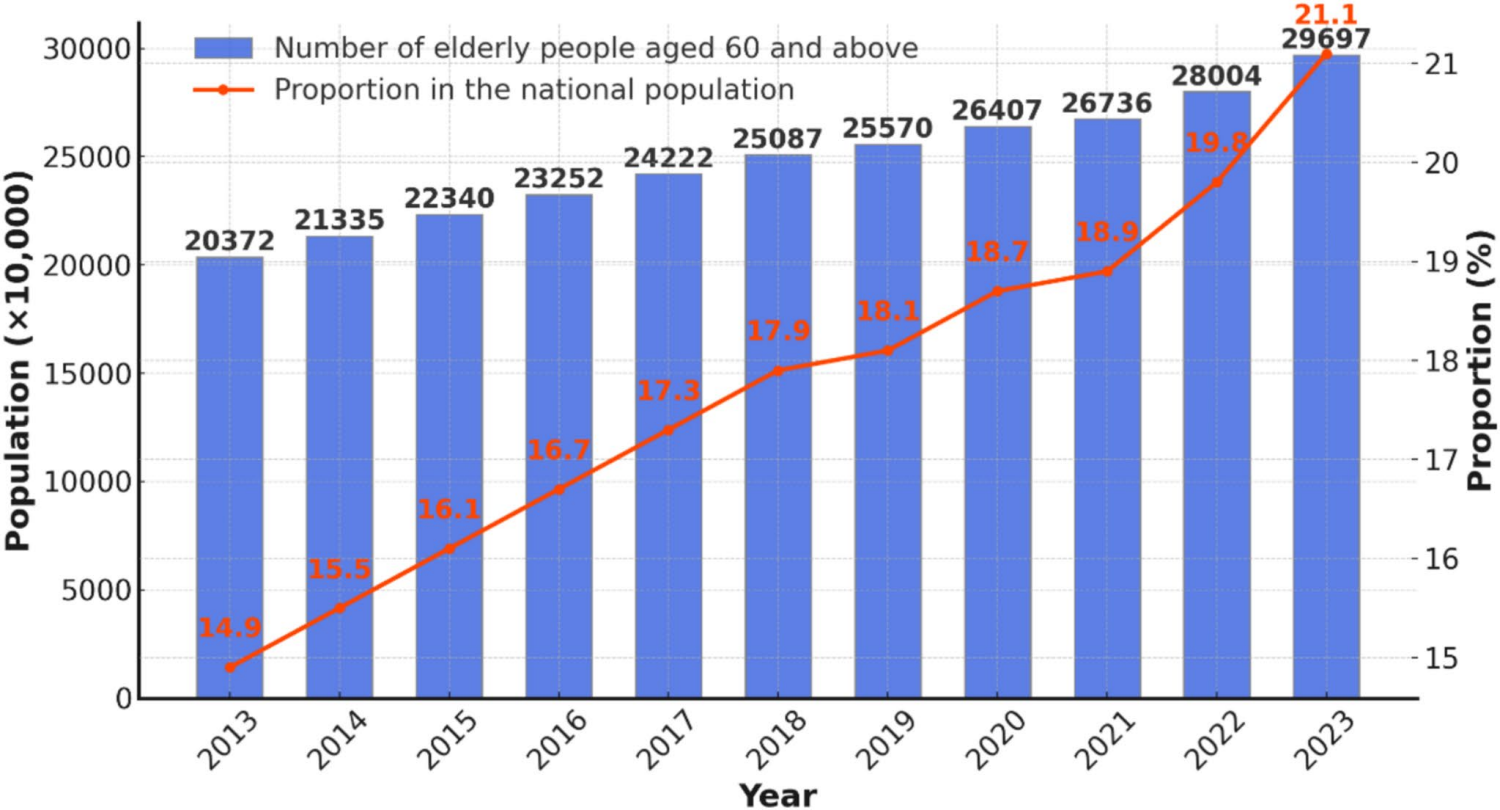
Number of older adult people over 60 years old in China and their proportion to the total population of the country by 2023.

China is undergoing rapid population aging at an unprecedented pace. According to United Nations standards, a country or region is classified as aging when the proportion of its population aged 65 and older exceeds 7%, deeply aging when it surpasses 14%, and super-aging when it exceeds 20% (13). Projections suggest that by 2050, China’s population aged 60 and older will reach 480 million, representing 35.1% of the total population, making it one of the most severely aging countries in the world (14). According to the 2023 National Bulletin on Aging Development, by the end of 2023, the older adult population aged 60 and above reached 296.97 million, comprising 21.1% of the total population (Figure 1). Additionally, 216.76 million individuals aged 65 and older comprise 15.4% of the total population (15).

The sixth national health service survey in China indicated that the two-week prevalence rate among the older adults was 55.5%, while the prevalence of chronic diseases reached 59.2% (16). In 2020, the population of disabled older adult individuals in China reached 52.71 million and is projected to surpass 77.65 million by 2030 (17). Older adult individuals are more vulnerable to diseases, endure prolonged illnesses, face multiple comorbidities, and recover more slowly due to declining physical functions (16).

CHSCs serve as the primary healthcare facilities for the older adults in China. Due to their physical vulnerability and increased need for medical care, proximity is critical for older adults (18). CHSCs, due to their proximity and convenience, provide the added advantage of integrating prevention and treatment, making them the preferred choice for the older adults (19). Statistics from the sixth National Health Service Survey indicate that 67.5% of residents with two-week illnesses seek care at primary healthcare centers, increasing to 69.4% among individuals aged 60 and above (20).

Projections indicate that China’s older adult population will reach 398 million by 2030. Based on the two-week consultation rate from the Sixth National Health Service Survey (2018), older adult individuals aged 60 and above are projected to require 4.15 billion medical consultations in 2030, presenting significant challenges to the healthcare system (17).

CHSCs in China face significant challenges due to their relatively recent establishment. According to the sixth National Health Service Survey, overall satisfaction with grassroots community health services in China is 84%, whereas satisfaction with the built environment is only 68.5%, significantly lower than general built environment satisfaction (16). Despite 91.8% of older adult individuals seeking treatment for illnesses, preventive and rehabilitative services remain severely lacking. Notably, only 1.9% of older adult individuals have received rehabilitative treatment, underscoring a critical gap in service provision (16).

These issues highlight significant shortcomings in CHSCs, particularly regarding their ability to meet the needs of the older adults. The disparities further emphasize the critical research significance of this study. Moreover, regional development imbalances across eastern, central, and western China, along with disparities in medical policies, have resulted in pronounced differences in the quality and accessibility of CHSCs. These disparities contribute to unequal access to essential medical services across regions.

Existing design standards for CHSCs often fail to align with current construction and operational realities. Outdated specifications do not adequately address the evolving demands of community health services, highlighting the need for urgent updates and adjustments (21). Furthermore, the service radius of many CHSCs is enormous, while inadequate location accessibility creates barriers for older adult individuals who rely on walking to access medical care.

### 1.2 Overview of CHSC

#### 1.2.1 Definition of the concept of CHSC

In the 1970s, the World Health Organization (WHO) introduced the concept of Community Health Service, and in 2006, the Chinese Ministry of Health issued the “Measures for the Administration of Urban Community Health Service Institutions,” which explicitly states: “Community health service institutions provide services to community residents, with a particular focus on the older adults, women, children, individuals with disabilities, and patients with chronic diseases, while also offering diagnostic and treatment services for common and chronic illnesses.” Community health service organizations prioritize community residents, with a particular focus on the older adults, women, children, individuals with disabilities, and those with chronic diseases. They provide diagnostic and treatment services for ordinary and prevalent illnesses while delivering rehabilitation, healthcare, prevention, and health education services (22).

Community health service organizations are centered around CHSCs, with community health service stations acting as supplementary units when necessary. Both serve similar purposes; however, CHSCs are larger, more versatile, and capable of delivering a broader range of services. As primary healthcare institutions in urban communities, CHSCs are vital in addressing key health issues and are integral to China’s healthcare system (23).

#### 1.2.2 Status of development of CHSC

As public health awareness rises, prevention, healthcare, and rehabilitation have become essential aspects of daily life. CHSCs, as the most accessible primary healthcare institutions, play an increasingly vital role in the lives of community residents. This increasing demand has driven improvements in the functionality of CHSCs, resulting in simultaneous growth in both their quantity and quality, contributing to the gradual development of an efficient, comprehensive, and accessible community health service system.

**Figure 2:**
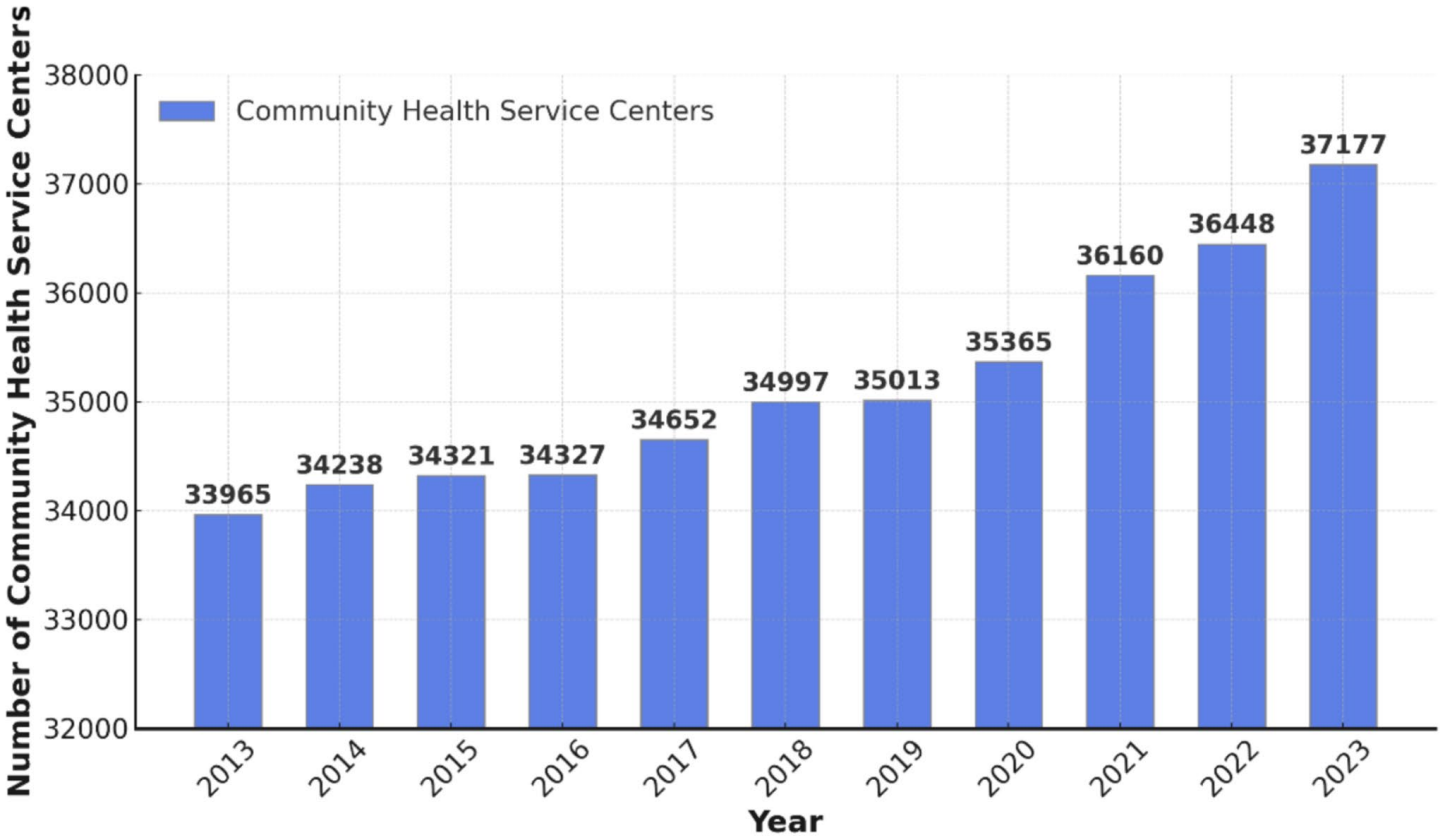
Number of CHSCs in China.

China’s healthcare system has undergone continuous development in recent years, which has been marked by a significant increase in CHSCs. According to the 2024 China Statistical Yearbook, China had 37,177 CHSCs in 2023, representing an increase of 729 from 2022 and a cumulative growth of 3,212 over the past decade (Figure 2) (24). Alongside community health service stations, CHSCs constitute core primary care resources, addressing the basic medical needs of residents and serving as a vital component of the hierarchical healthcare system (25).

#### 1.2.3 Classified treatment system

China’s hierarchical diagnosis and treatment system categorizes medical services into first-, second-, and third-level institutions based on illness severity, urgency, and treatment complexity, forming a three-tiered framework for healthcare services. The “Measures for the Hierarchical Management of Hospitals” categorize hospitals by their functions and responsibilities: First-level hospitals focus on preventive care, healthcare services, medical treatment, and rehabilitation for community residents, addressing basic medical needs and relying primarily on primary healthcare organizations and health centers. Second-level hospitals provide comprehensive medical services to multiple communities or regions and engage in teaching and scientific research, primarily within regional hospitals. Third-level hospitals offer advanced, comprehensive medical services at the national, provincial, and municipal levels, playing a key role in higher medical education and scientific research, primarily within large general hospitals (Table 1) (26).

**Table 1:** China’s hierarchical diagnosis and treatment system.

Hierarchy	Services	Service level	Medical organization
First-level	Preventive, medical, health, and rehabilitation services	Communal	Primary care institutions, health centers
Second-level	Comprehensive medical and health services, teaching, research, and development.	Multiple communities, regions	Regional hospitals
Third-level	High-level comprehensive medical care, higher education, and research missions	Cross-regional, cross-provincial, national	Hospitals above the regional level

The hierarchical diagnosis and treatment system directs patients with chronic and common diseases to seek initial care at primary healthcare facilities, providing residents greater convenience, reduced costs, and higher reimbursement rates, thus alleviating financial burdens and saving time (27). For more complex cases, patients are referred to second or third-level hospitals for specialized treatment. This system effectively diverts patient flow, reduces pressure on large general hospitals, optimizes medical resource allocation (28), and supports the model of “seeking care at the community level for minor illnesses and hospitals for major illnesses.”

This study focuses on CHSCs, community-based primary care facilities that operate as part of first-level hospitals.

### 1.3 Purpose of the study

This study aims to evaluate the cyclability and walkability of CHSCs across the eastern, central, and western regions of China, systematically assess the equity of their distribution, and clarify the current state of healthcare resource distribution among these regions. The study aims to provide a scientific basis for optimizing the spatial distribution of CHSCs and improving their accessibility, thereby promoting the equalization and equity of public health services, supporting China’s public health policies in response to population aging, and contributing to the realization of the “Healthy China 2030” initiative.

## 2 Materials and methods

### 2.1 Scope of the study

According to the 2024 China Statistical Yearbook, the number of CHSCs in China reached 37,177 by the end of 2023. However, CHSC development in China remains in its early stages, characterized by significant regional disparities, with many centers underdeveloped and lacking sufficient research value. Therefore, conducting a comprehensive analysis of all CHSCs is impractical and unnecessary. To enhance the relevance and practicality of the study, this paper focuses on state-designated exemplary CHSCs as the research subject.

In 2017, the National Health and Family Planning Commission convened experts to identify 203 National Excellent Community Health Service Centers (CHSCs), using the Community Health Service Quality Evaluation Indicator System (2015 Edition) as the selection criterion. These centers exhibit notable service quality and management strengths, rendering them valuable research subjects.

**Figure 3:**
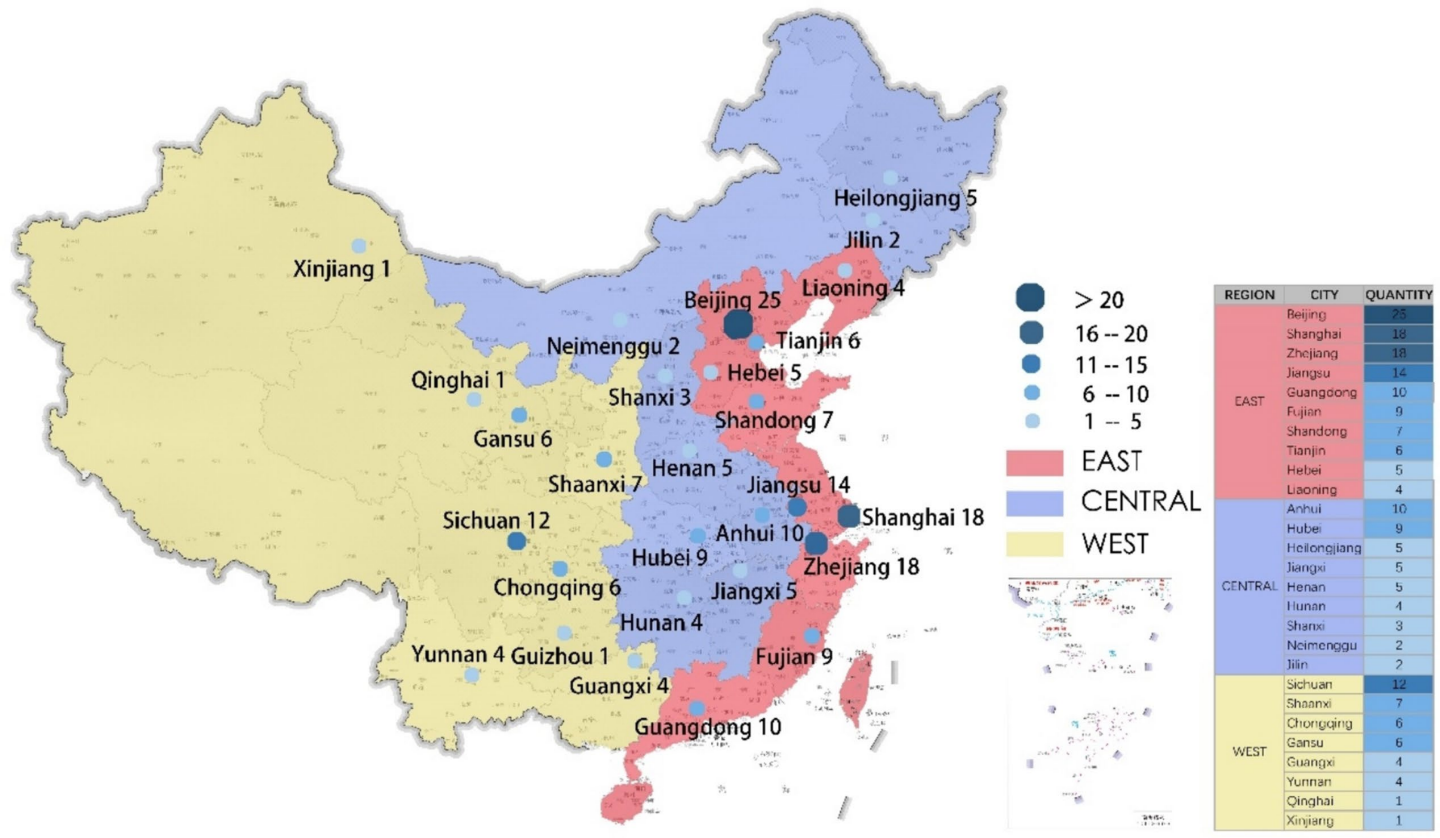
Distribution map of outstanding CHSC in China.

This study focuses on National Excellent CHSCs, which are distributed across 78 cities nationwide (Figure 3). 116 centers are situated in the eastern region across 33 cities, 45 in the central region across 27 cities, and 42 in the western region across 18 cities (Table 2). To enhance the study’s precision, 110 CHSCs from 15 cities were selected as the sample, representing the top five cities in the eastern, central, and western regions.

**Table 2:** Statistics on the number of outstanding CHSCs in different regions.

Region	Number of cities	Number of outstanding CHSC	The number of cities selected for this study	The number of CHSCs was chosen for this study
East	33	116	5	63
Central	27	45	5	20
Western	18	42	5	27
Subtotal	78	203	15	110

### 2.2 Research methodology

#### 2.2.1 GIS-based cyclability indicators

Geographic Information Systems (GIS) is an interdisciplinary field that integrates geography, cartography, remote sensing, and computer science and is extensively applied across various domains (29). GIS is a computer-based tool with powerful computational and analytical capabilities, facilitating scientific management and intuitive data visualization through spatial information processing. In recent years, GIS technology has seen growing application in urban planning, particularly for calculating cyclability, with its scientific validity substantiated by numerous studies (30). GIS methods based on road network analysis identify residents’ actual reachable paths by bicycle rather than relying solely on a simple circular radius (31).

The cyclability indicator typically evaluates residents’ ease of access to Community Health Service Centers (CHSCs) by bicycle within a specific area. This study utilizes GIS technology to integrate neighborhood road networks, the spatial distribution of CHSCs, and residential district locations. By urban planning standards for public service facilities, a 1,000-meter coverage radius is adopted as the cyclability indicator. The 1,000-meter service radius reflects the maximum distance residents can comfortably cycle under typical conditions. A 1000-meter distance is generally regarded as reachable within 5 min by bicycle, ensuring convenient access to essential medical services and significantly improving community health service cyclability (32).

Buffer analysis is a fundamental GIS technique used to evaluate the spatial coverage of community health service centers (CHSCs) by bicycle. Creating buffer zones of specific distances (e.g., 1 kilometer, 3 kilometers) around each CHSC can quantify the cyclability of healthcare services. Cyclability is defined as the proportion of residents within the buffer zone relative to the total population in the study area (Equation 1).

Formula:


(1) $$\mathrm{Cyclability}=\frac{N_{\mathrm{buffer}}}{N_{\mathrm{total}}}\times 100\%$$

Where:

*N_bufrer_*: The number of residents within the buffer zone was calculated using spatial overlay analysis in GIS.

*N_total_*: The total number of residents in the study area is derived from population data integrated into the GIS environment.

#### 2.2.2 Walkability indicators based on the walk score

As global urbanization progresses, urban transportation infrastructure is becoming increasingly advanced. However, in many cities, road designs prioritize motorized transportation, often at the expense of pedestrian needs. The widespread use of motorized vehicles contributes to environmental pollution and diminishes the necessity for walking, negatively affecting public health (33). Walking, as a low-carbon and eco-friendly mode of transportation, provides substantial ecological, social, and economic benefits and significant health advantages for urban residents. Consequently, fostering a walkable environment holds critical social significance (34).

Walkability is a spatial attribute reflecting how the built environment influences individuals’ decisions to walk, encompassing factors such as proximity between origins and destinations and the comfort and convenience of the walking experience (35). The theory of walkability emerged after World War II, coinciding with advancements in automobile technology. Amid the rapid expansion of modernist cities, people began recognizing the challenges associated with large-scale urban planning. In 1961, Jane Jacobs critically examined American urban development in The Death and Life of Great American Cities, advocating for the integration of sidewalks and streets, profoundly influencing subsequent urban planning (36).

In the early 1990s, as automobile use became widespread, American cities encountered escalating challenges. This shift redirected attention toward urban development models focused on intensification, efficiency, and greening, giving rise to “New Urbanism.” The community theory underpinning New Urbanism emphasizes diversity, walkability, compactness, and functional integration, with the “5-min walking distance” model as a core concept (37).

By the late 1990s, transportation research in the United States further refined the concept of walkability, emphasizing the built environment’s influence on walking behavior (38). In 2000, the United States, the United Kingdom, Canada, Australia, and 19 other countries initiated studies on walkability. That same year, these nations jointly founded the “21st Century Walkers Association” to promote walkable communities.

In 2003, the United Kingdom introduced the “low-carbon” theory, reaffirming walkability as a form of green transportation. That same year, the introduction of the “Community Environmental Walkability Scale” initiated the quantitative phase of walkability research (39, 40). In 2007, the United States launched the “Walk Score” website,^1^ quickly establishing it as a widely used tool among government officials, planners, and stakeholders. Since then, it has remained a key reference for government officials, planners, and researchers.

With the growing significance of walkability, quantitative measurement methods have undergone extensive development. Walkability measurement methods are broadly categorized into two main types: those focusing on community destinations, highlighting the convenience of residential locations in accessing functional destinations, and those emphasizing the walking environment, including the livability and safety of streets (41). American researchers introduced the Walk Score concept in 2017 in response to the first approach. This method evaluates public service facilities within residential areas as destinations, integrates road network data, and accounts for factors such as distance decay, intersection density, and block length, thereby improving the scientific precision of the results. Since then, this method has gained widespread adoption among international researchers (42, 43).

Two primary algorithms are used to calculate walk scores: single-point and surface walk scores. The single-point walk score calculates walkability based on specific origin and destination points, yielding highly accurate measurements. In contrast, the surface area walk score applies to more significant regions, such as neighborhoods, communities, or cities, representing the overall walkability of the area (44). This study applies the single-point walkability index due to the community scale’s clearly defined residential and amenity points and a relatively complete open road network. This method ensures data accuracy and aligns well with the characteristics and requirements of the research.

The calculation of the single-point walk score relies on two primary data sources: (1) open road network data detailing the road network’s layout, intersection density, and neighborhood length, and (2) origin and destination data, typically obtained from open maps. In this study, Point of Interest (POI) data from the Little O map is the basis for calculation.

Calculating a single-point walk score involves three key steps. First, a classification table of facilities is developed, with weights assigned according to the type of facility being assessed. Second, the base walk score is derived using the principle of walking distance decay. Finally, adjustments are made to the data based on intersection density and block length. The walk score is then normalized to a scale of 0–100 to produce the final value (45).

The walk score for a single point is calculated based on the weighted sum of facilities within the walking range, considering the distance decay effect. The formula is as follows (Equation 2):


(2) $$S=h\left(a,b\right)\overset{N}{\underset{n=1}{\sum }}\left(a_{n}\cdot b_{n}\right)$$

Where:

*S*: Walk score for the single point.

*a_n_*: Weight of facility *n*, representing its importance or contribution to walkability.

*b_n_*: Distance decay coefficient for facility *n*, reflecting the impact of distance on accessibility.

*N*: Total number of facility types within the walking range.

*h(a, b)*: A function that combines the weights and distance decay coefficients.

The base walk score calculation primarily considers the diminishing effect of distance on walking behavior. Generally, the willingness to walk declines as the distance between origin and destination increases. This pattern is often modeled through a multi-curve approach. At a standard walking speed of 4.8 km/h, a 5-min walk equals 400 meters, a 20-min walk to 1,600 meters, and a 30-min walk to 2,400 meters. Walking is generally not selected as a mode of transportation when distances exceed 2,400 meters.

**Figure 4:**
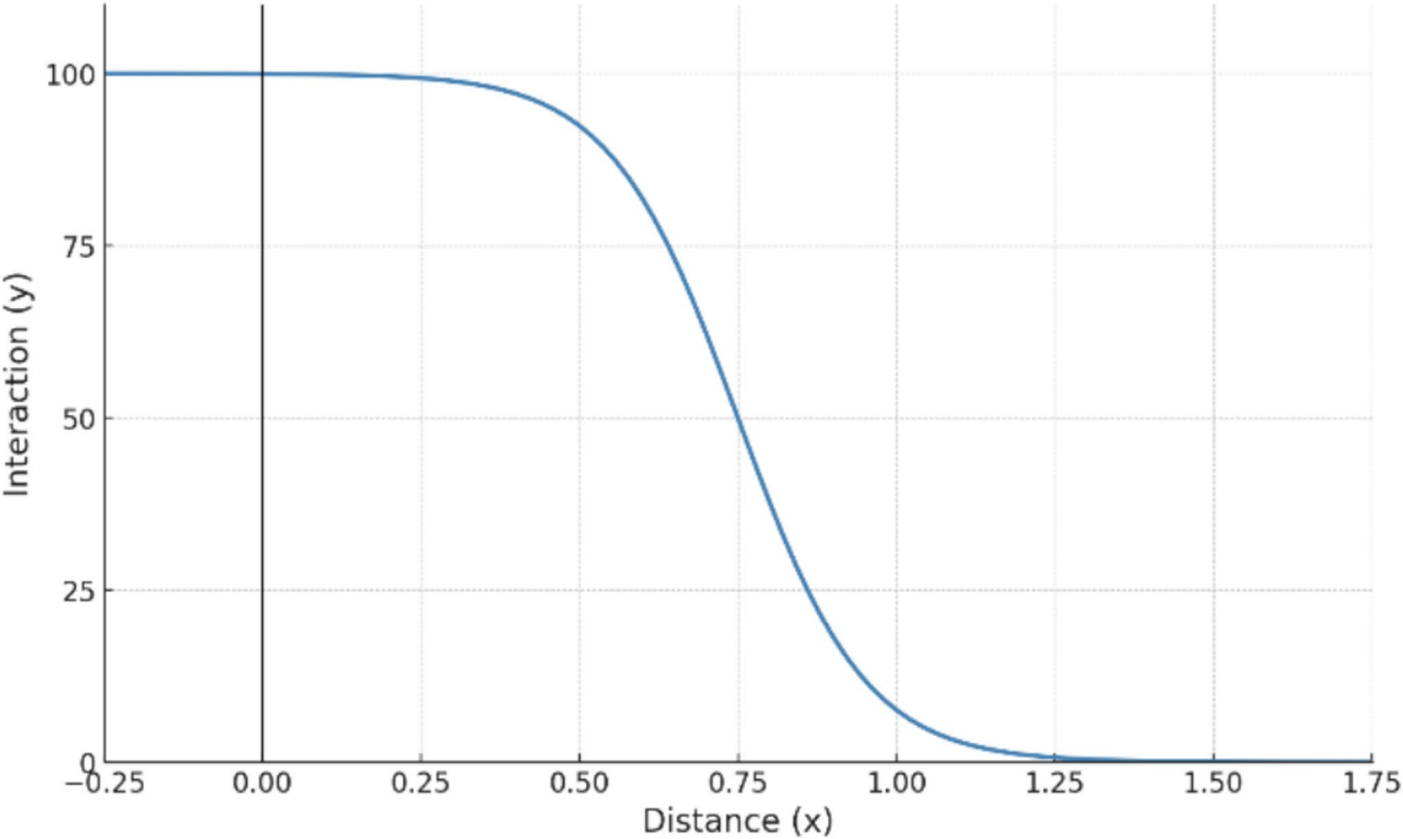
Distance decay curve.

According to the walking attenuation model (Figure 4), no attenuation occurs within 400 meters. Rapid attenuation begins beyond 400 meters, reaching 12% at 1,600 meters. Beyond 1,600 meters, attenuation slows, and for distances exceeding 2,400 meters, the attenuation rate surpasses 1, yielding a walk score of 0 (35).

The base walk score is adjusted according to existing studies to improve data accuracy. This adjustment primarily relies on two key factors: road intersection density and neighborhood size (46). Higher road intersection density improves walking path accessibility, reducing the attenuation rate. Similarly, smaller neighborhood sizes enhance walkability, lowering the attenuation rate. Conversely, low road intersection density or large neighborhood size reduces walking convenience and comfort, increasing the attenuation rate.

The combined effect of these factors caps the maximum attenuation rate adjustment at 10% (Table 3). This adjustment method more accurately reflects the impact of road and neighborhood conditions on walking, aligning the calculated walk score with real-world situations.

**Table 3:** Road intersection density and intercept length decay rate.

Density of intersections/(units/km2)	Attenuation index	Block length/m	Attenuation index
(77, +∞)	0.00	[0, 120]	0.00
(57, 77)	0.01	[120, 150]	0.01
(47, 57)	0.02	[150, 165]	0.02
(35, 47)	0.03	[165, 180]	0.03
(23, 35)	0.04	[180, 195]	0.04
(0, 23)	0.05	[195, +∞]	0.05

This study applies the single-point walk score calculation method with GIS tools. First, open road network data, CHSC locations, and residential area coordinates were extracted from open maps. Next, the walk score from each settlement point to every healthcare facility was calculated using the walking distance decay model. The average walk score for all facilities was computed to derive the walkability index at each settlement point. Finally, the walkability indices of all residential points were averaged to determine the overall community walkability index, reflecting pedestrian accessibility to community health facilities.

### 2.3 The calculations of CHSC cyclability indicators and walkability indicators

The following example illustrates the calculation of cyclability and walkability indicators for a CHSC in Desheng Street, Xicheng District, Beijing. Desheng Street, situated in Xicheng District, Beijing, lies between the North Second and North Third Ring Roads. It extends 2.1 km from east to west and 2.7 km from north to south, encompassing a total area of 4.1 square kilometers. As of June 2020, Desheng Street administered 20 communities.

Eight CHSCs serve the community. These centers provide a broad range of services to Desheng community residents, including basic medical care, preventive healthcare, family planning consultations and services, health education, and rehabilitation support.

**Figure 5:**
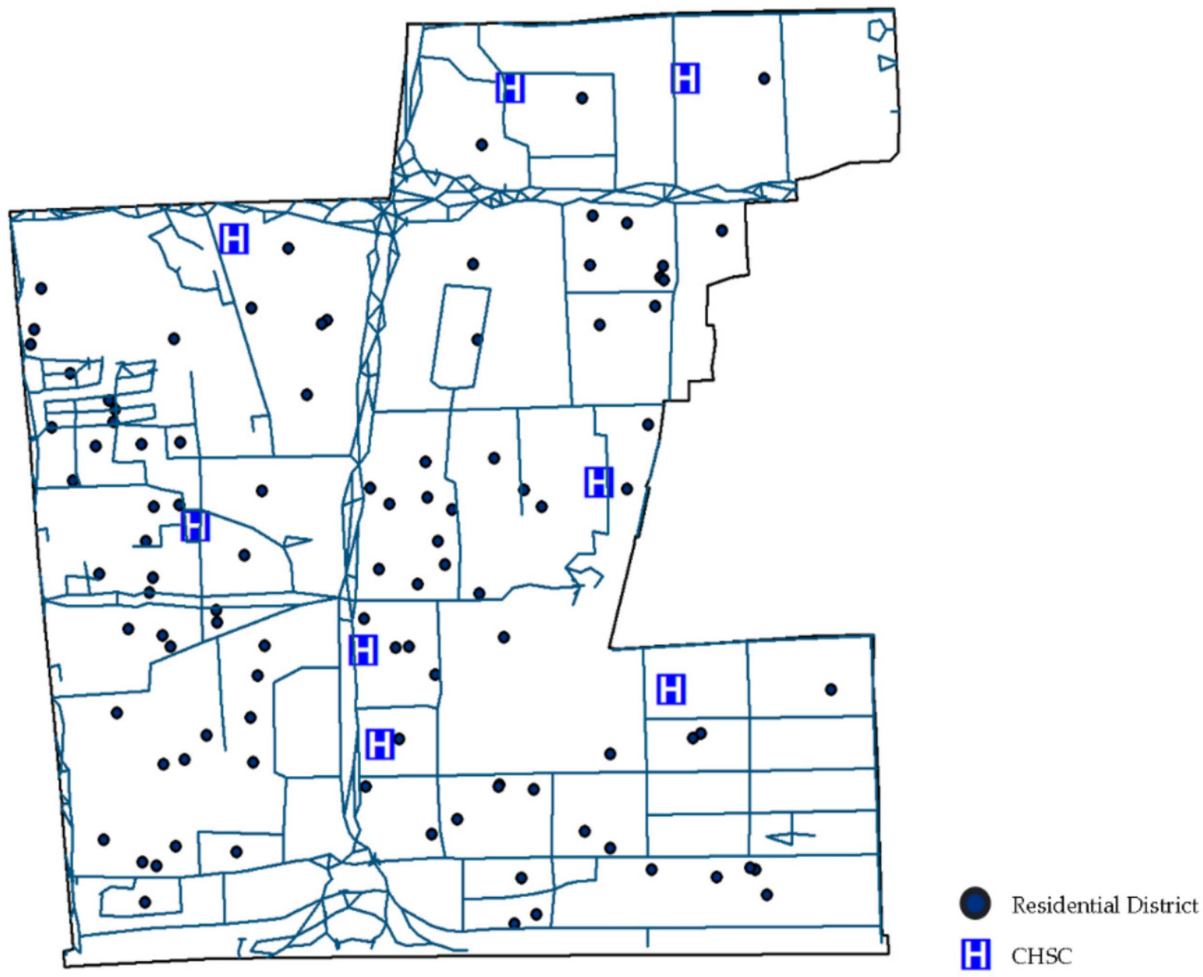
Road network and POI data for the Deschutes community.

**Figure 6:**
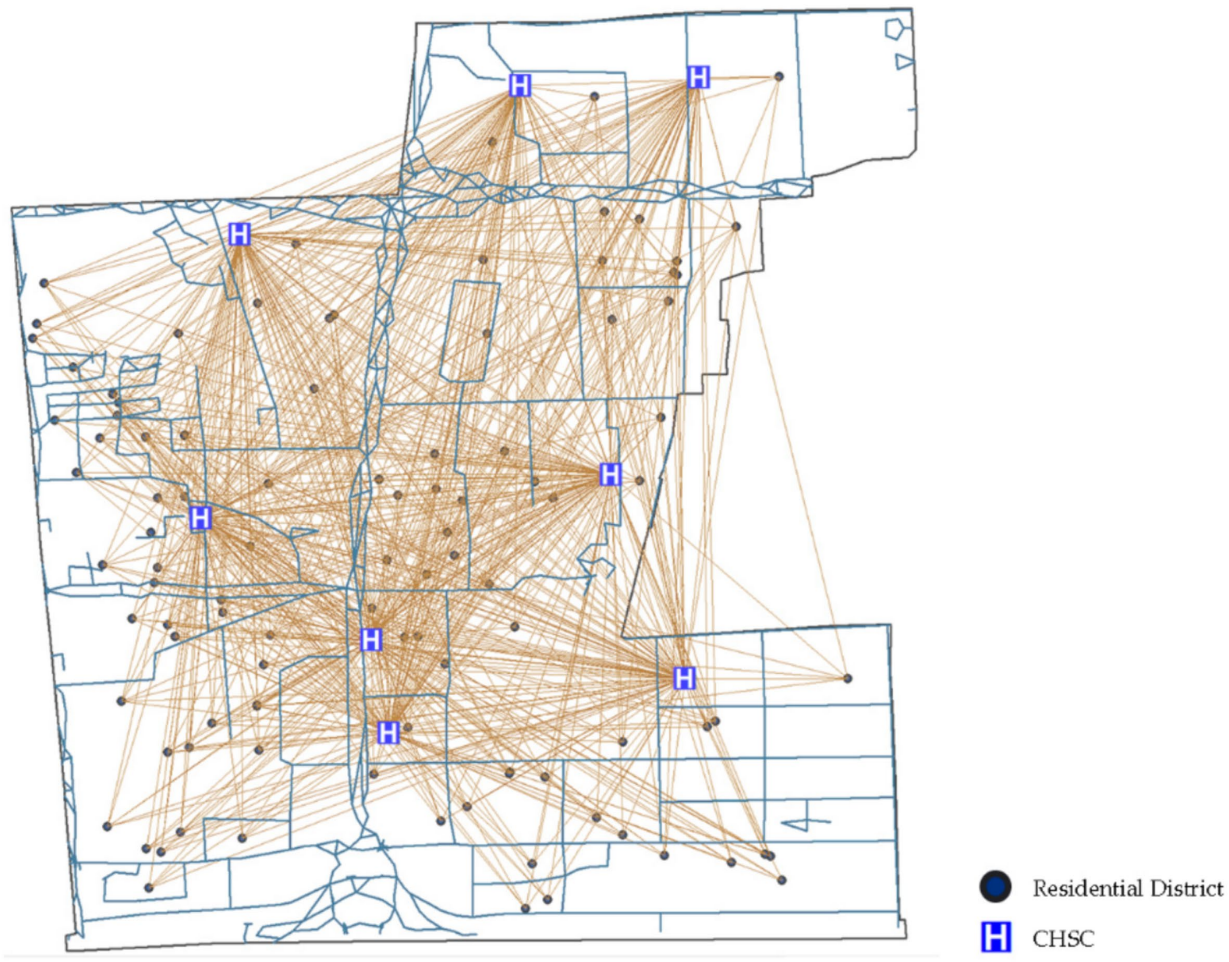
Calculation of walkability indicators for CHSC.

POI data from Little O Maps identifies 100 residential neighborhoods within the community (Figure 5). The community was analyzed using GIS-based walkability indicator calculations (Figure 6).

This study applied the single-point walk score method to calculate the walkability index. First, open road network data, CHSC points, and residential neighborhood points were extracted from open map sources. The average walkability index for all residential points was then calculated using the walking distance attenuation model, resulting in a walkability index of 0.403 for accessing community health service facilities on foot.

**Figure 7:**
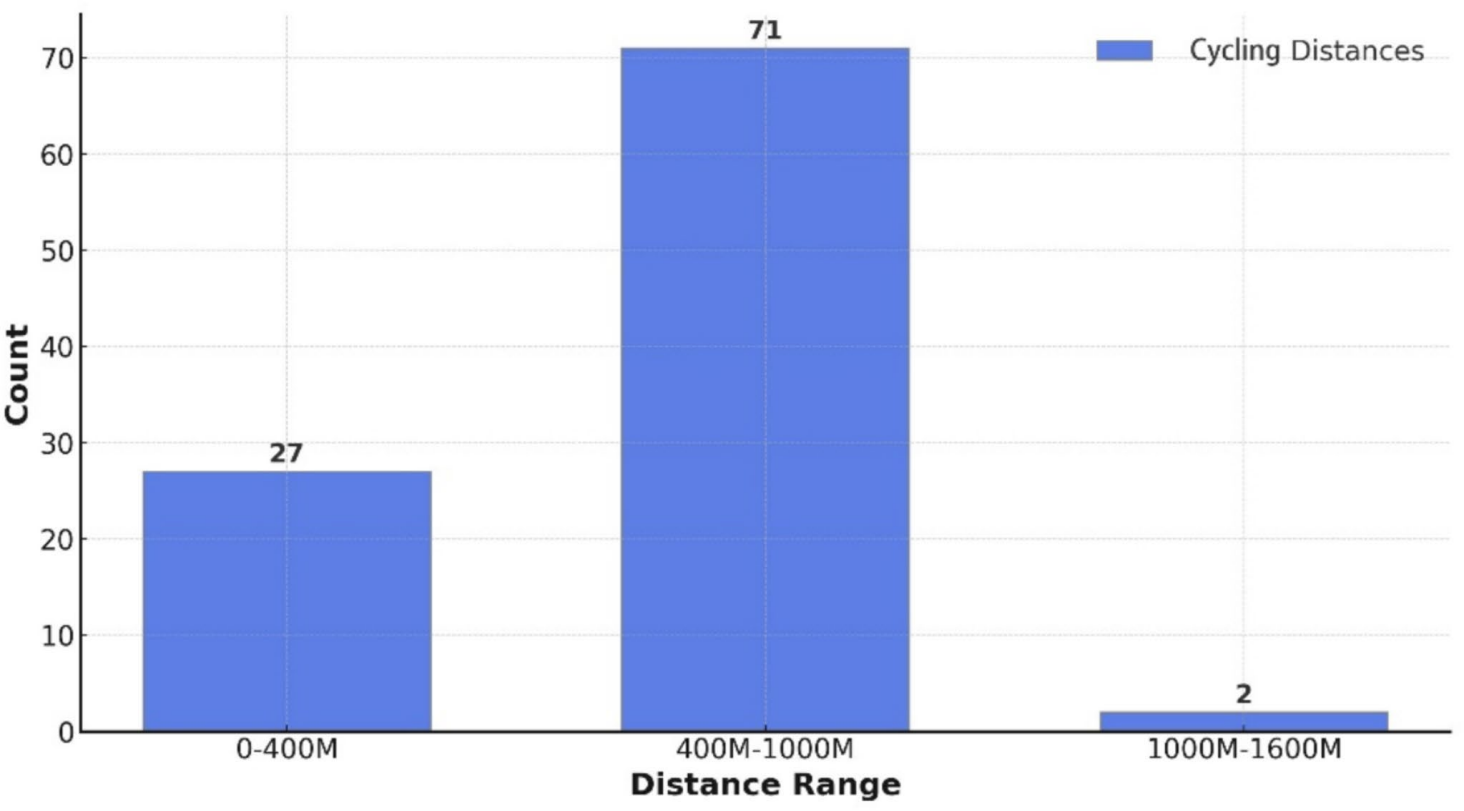
Cycling distances to residential areas within the Desheng community.

Cyclability indicators were calculated, showing that 27 residential areas had a cycling distance of less than 400 meters, 2 exceeded 1,000 meters, and 71 fell between 400 and 1,000 meters. Over 98% of residential areas can access the CHSC by bicycle within 1,000 meters (Figure 7), meeting the 1,000-meter service radius requirement established by the Urban Residential Area Planning and Design Standards. This demonstrates that the Desheng community’s cyclability indicators meet the fundamental requirements specified by the code.

The cyclability and walkability indicators for 110 CHSCs across the eastern, central, and western regions were calculated using the GIS-based algorithm described above.

## 3 Results

### 3.1 Cyclability and walkability of CHSC in the eastern region

**Figure 8:**
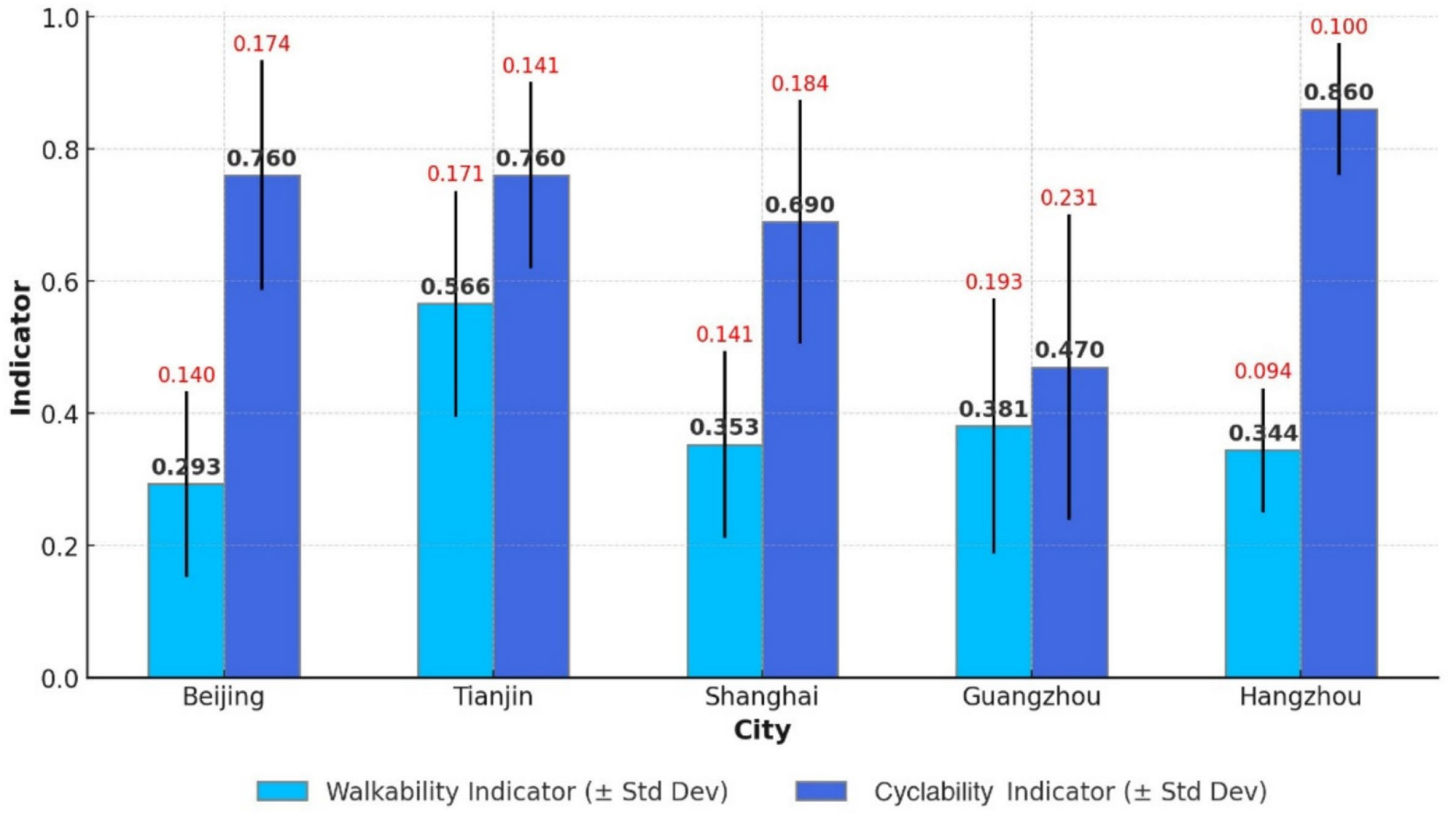
Walkability and cyclability of CHSC in five cities in the eastern region.

A comparison of five eastern cities shows that the mean walkability indicator is 0.293 for Beijing (SD = 0.14), 0.566 for Tianjin (SD = 0.171), 0.353 for Shanghai (SD = 0.141), 0.381 for Guangzhou (SD = 0.193), and 0.344 for Hangzhou (SD = 0.094). The average walkability for the eastern cities is 0.351. Based on these findings, the community health service walkability ranking from highest to lowest is Tianjin > Guangzhou > Shanghai > Hangzhou > Beijing (Figure 8). Notably, the walkability indicators remain relatively low.

An analysis of the 1,000-meter CHSC coverage rates by bicycle in the five cities reveals the following: 0.76 in Beijing, 0.69 in Shanghai, 0.86 in Hangzhou, 0.47 in Guangzhou, and 0.76 in Tianjin. The average cyclability indicator for the eastern region is 0.71. None of the cities achieved full 1,000-meter coverage. For 1,000-meter cyclability to community health services, the cities rank from highest to lowest, such as Hangzhou > Beijing = Tianjin > Shanghai > Guangzhou (Figure 7).

### 3.2 Cyclability and walkability of CHSC in the central region

A comparison of walkability indicators in five central Chinese cities reveals the following: Wuhan (mean = 0.31, SD = 0.263), Hefei (mean = 0.39, SD = 0.101), Harbin (mean = 0.629, SD = 0.328), Zhengzhou (mean = 0.549, SD = 0.07), and Changsha (mean = 0.131, SD = 0.071). In descending order, the community health service walkability in these five cities is ranked as Harbin > Zhengzhou > Hefei > Wuhan > Changsha, with an average of 0.388 for central cities (Figure 8). Compared to the eastern region, walkability indicators in central China are higher.

**Figure 9:**
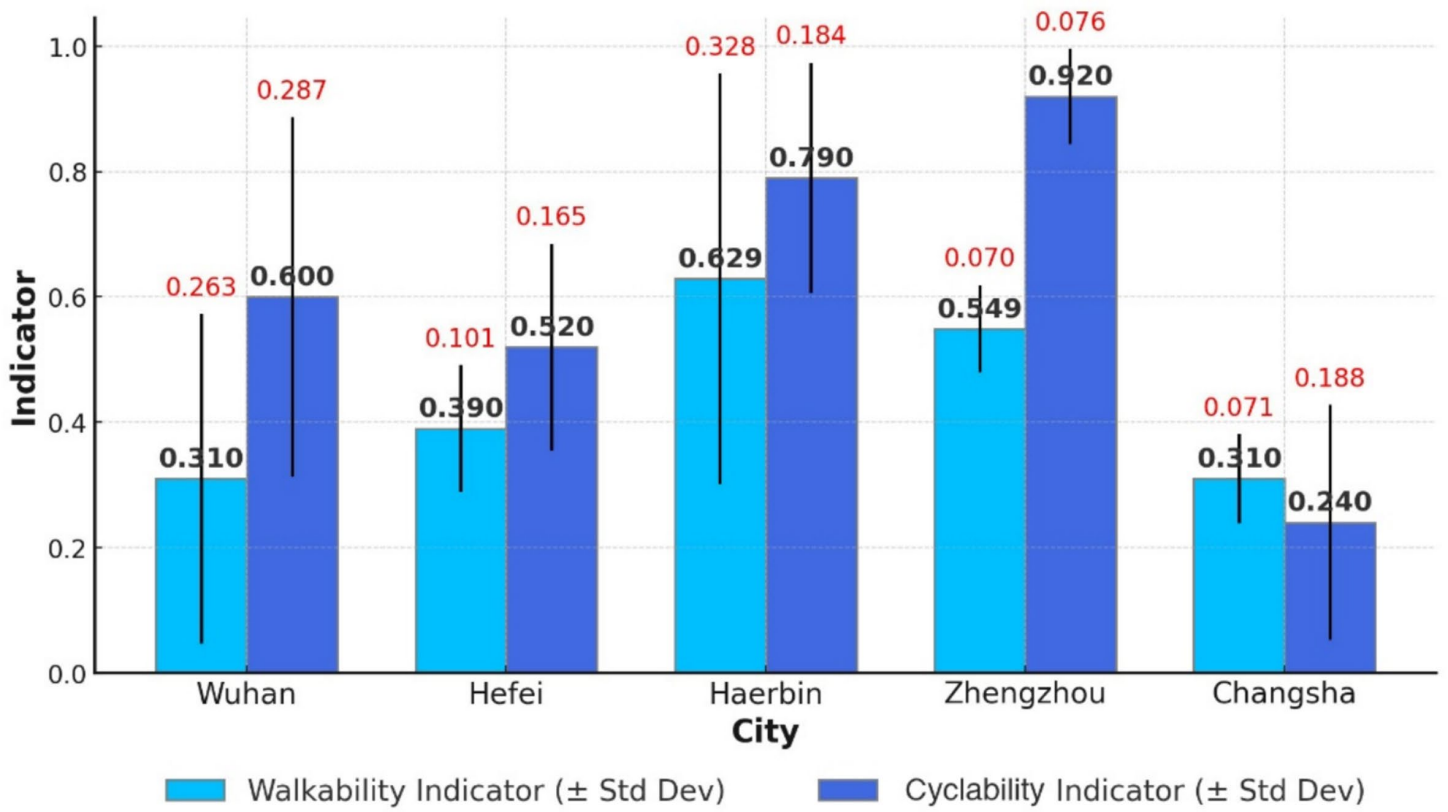
Walkability and cyclability of CHSC in five cities in the central region.

An analysis of the 1,000-meter CHSC coverage rates by bicycle in five central cities shows the following: Wuhan (0.60), Hefei (0.62), Harbin (0.79), Zhengzhou (0.92), and Changsha (0.24). None of the cities achieved full 1,000-meter coverage, with an average rate of 0.64, indicating that only 64% of settlements have access to a CHSC within this distance by bicycle. Cyclability in the central region is lower than in the eastern region. The 1,000-meter cyclability of community health services in these five cities, ranked from highest to lowest, is Zhengzhou > Harbin > Hefei > Wuhan > Changsha (Figure 9).

### 3.3 Cyclability and walkability of CHSC in the Western region

The mean walkability indicator values for five western cities are: Chongqing (0.323, SD = 0.195), Chengdu (0.261, SD = 0.179), Kunming (0.123, SD = 0.041), Xi’an (0.551, SD = 0.261), and Guilin (0.124, SD = 0.035). A comprehensive analysis ranks walkability for community health services as Xi’an > Chongqing > Chengdu > Guilin > Kunming, with an average score of 0.287 for the five western cities (Figure 9). The western region exhibits the lowest walkability indicators among the three regions.

**Figure 10:**
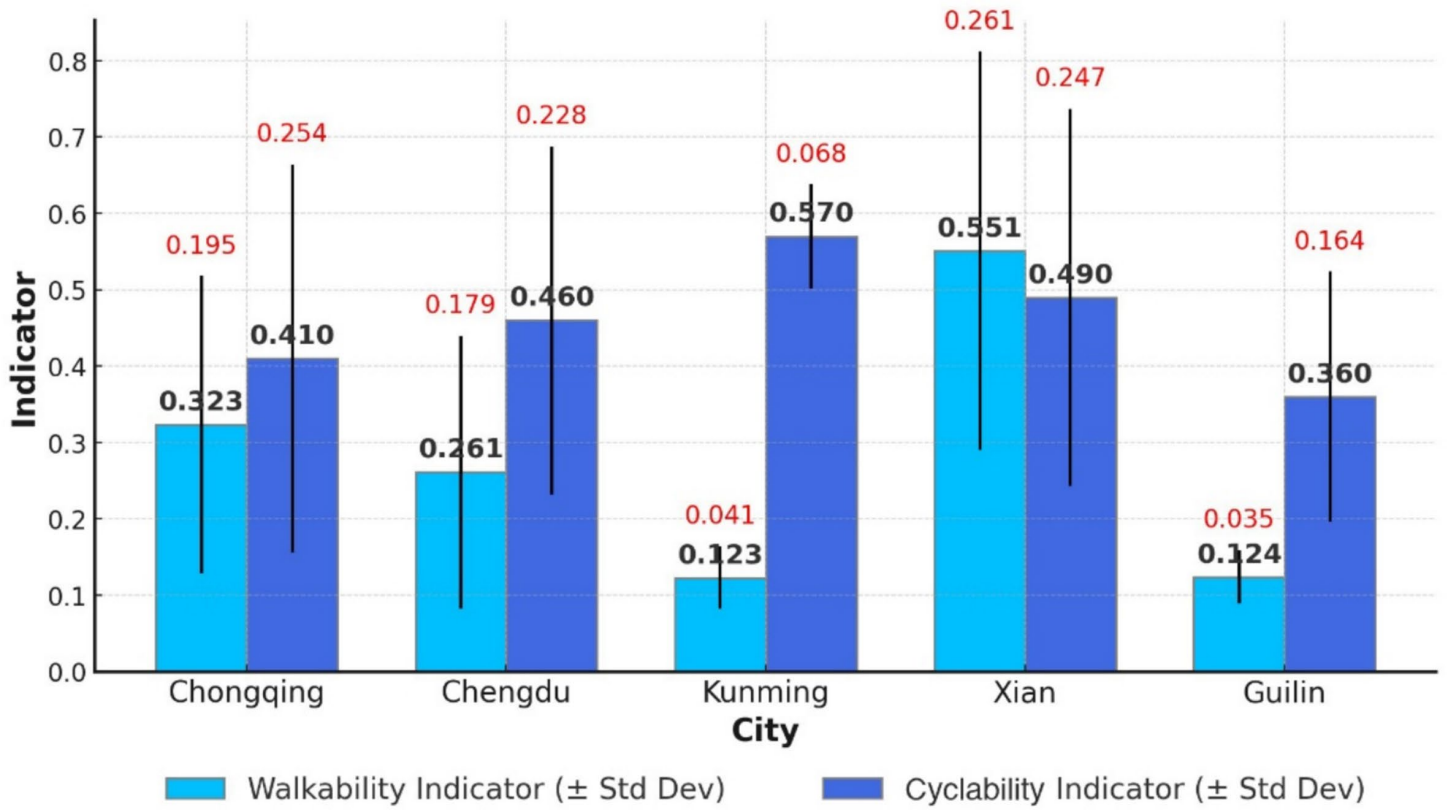
Walkability and cyclability of CHSC in five cities in the western region.

An analysis of 1,000-meter CHSC coverage by bicycle in five cities shows the following rates: Chongqing (0.41), Chengdu (0.46), Kunming (0.57), Xi’an (0.49), and Guilin (0.36). Except for Kunming, the 1,000-meter cyclability in the other four cities remains below 0.5. The five cities ranked in 1,000-meter cyclability are as follows: Kunming > Xi’an > Chengdu > Chongqing > Guilin (Figure 10). The average 1,000-meter cyclability for the five cities is 0.46, significantly lower than that of the eastern and central regions.

### 3.4 Comparison of walkability and cyclability in the east, center, and west

Table 4 shows that the 1,000-meter cyclability indicators for the East, Central, and West are 0.71, 0.64, and 0.46, respectively. The average facility density indicators, measured in units per square kilometer, are 1.196 for the East, 1.115 for the Central region, and 0.681 for the West. The East outperforms the other regions, while the West performs poorly.

**Table 4:** Comparison of indicators for the east, center, and west.

Indicator	East	Central	Western	Comparative radar maps by region
Walkability	0.351	0.388	0.287	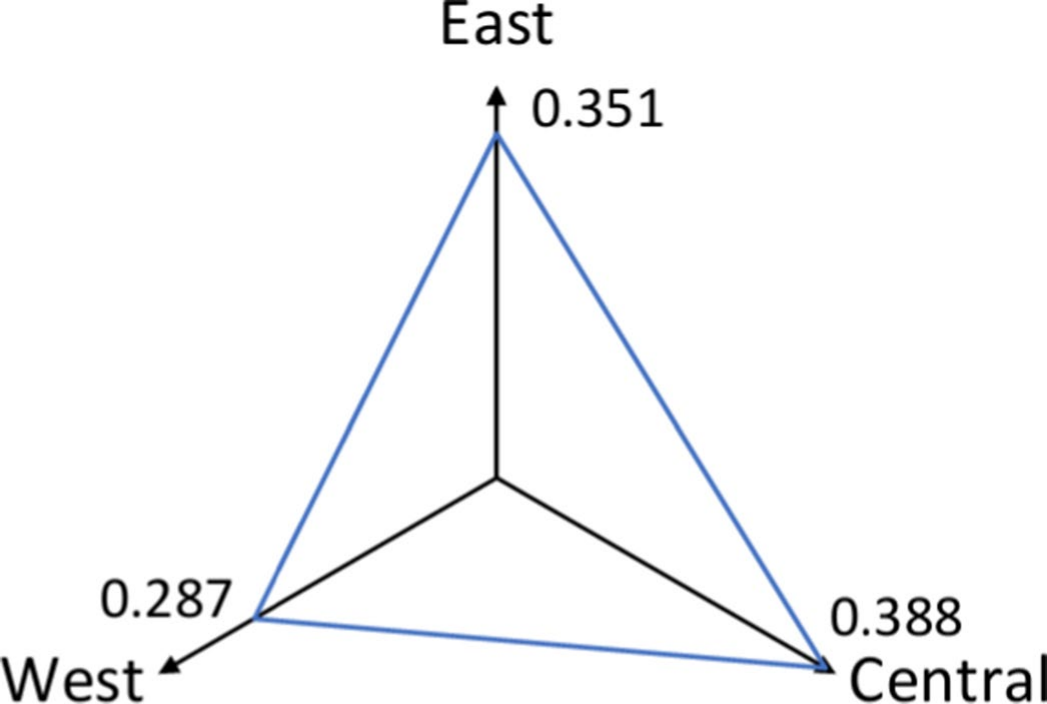
Cyclability	0.71	0.64	0.46	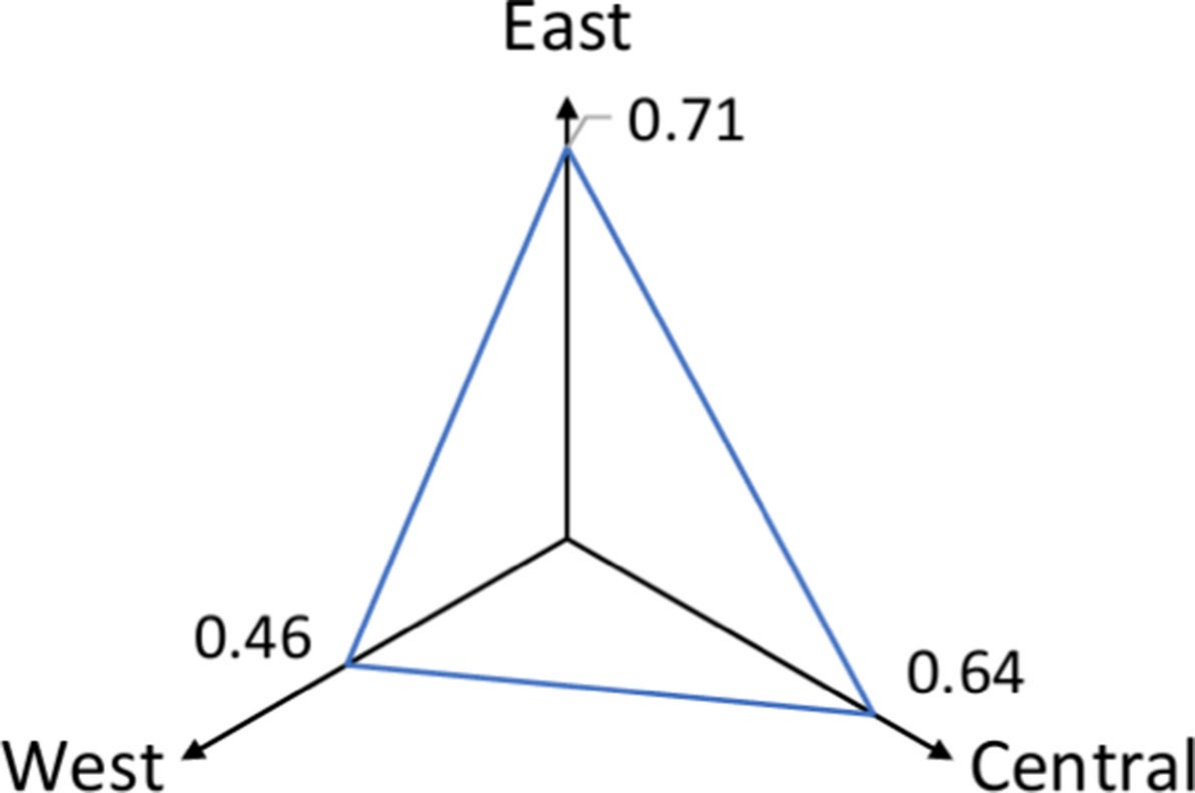
Average facility density	1.196	1.115	0.681	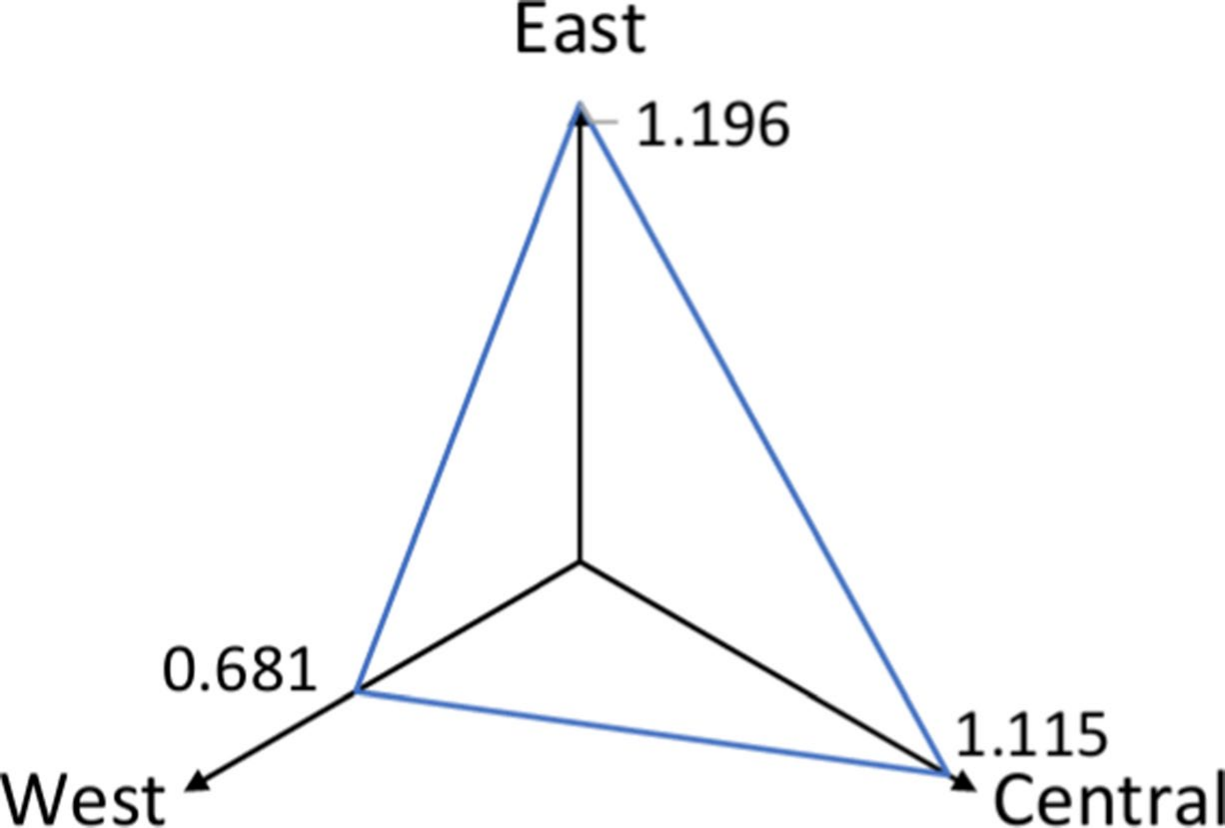

None of the regions—East, Central, or West—achieved full 1,000-meter settlement coverage by bicycle, falling short of the government’s service radius requirement. Walkability indicators for the East, Central, and West regions are 0.351, 0.388, and 0.287, respectively. Based on walkability index criteria, all regions show relatively low walkability, with the East and Central regions outperforming the West.

Despite lower cyclability and facility density, the Central region shows slightly better walkability than the East. This suggests that CHSC planning and layout in the Central region is more effective than in the East.

An analysis of contributing factors shows that the Eastern and Central regions benefit from advanced urbanization, economic development, and superior infrastructure, including better roads and healthcare facilities. This leads to greater overall cyclability and walkability. In contrast, the Western region, which developed later, continues to lag in cyclability and walkability, although cities like Chengdu and Chongqing show relatively more substantial community healthcare development.

## 4 Discussion

This study examines the equity of Community Health Service Centers (CHSCs) across eastern, central, and western China through 110 exemplary cases, focusing on two key dimensions: cyclability and walkability. The findings reveal significant differences in the spatial layout and service capacity of CHSCs, highlighting regional disparities in older adult accessibility. Given the increasing proportion of older adults in China, these disparities raise important concerns about equitable access to primary healthcare for aging populations, which is essential for managing chronic conditions and promoting healthy aging.

### 4.1 Service radius coverage

The Eastern region performs best in terms of 1,000-meter service radius coverage (0.71), followed by the Central region (0.64) and the Western region, with the lowest coverage (0.46). This suggests that CHSCs in most Chinese cities fall short of the government’s 1,000-meter service radius target. The Western region’s coverage rate, below 0.50, highlights a critical shortage of community healthcare facilities, directly affecting access to essential healthcare services, especially for older residents who may have limited mobility or transportation options. Ensuring service radius coverage is particularly important for this demographic, as proximity greatly influences their ability to seek timely and continuous medical care.

### 4.2 Walkability indicators

Walkability indicators across all regions are significantly below the ideal value, reflecting poor facility walkability. The Eastern and Central regions outperform the Western region, indicating that higher urbanization levels, better road networks, and more evenly distributed medical facilities provide greater convenience in these regions. For older adults, walkable access to healthcare is not just a matter of convenience but a determinant of health equity and autonomy. Insufficient walkability may discourage healthcare utilization among older adult individuals, especially those with functional limitations. The Western region, with lower indicators, should prioritize expanding the number of CHSCs in future developments.

### 4.3 Implications for policy and practice

The analysis highlights significant CHSC cyclability and walkability deficiencies across China, with the Western region experiencing the most pronounced gaps. This suggests that CHSCs, as fundamental healthcare facilities in China, are insufficient in number and unevenly distributed across regions. Policymakers should prioritize expanding CHSC coverage in underserved areas, particularly in the Western region, and improving cyclability and walkability through urban planning and infrastructure development. These improvements are particularly urgent for aging populations, who are more dependent on local healthcare services. Enhancing active accessibility for the older adults can help reduce health disparities, improve disease prevention, and support the development of age-friendly communities.

## 5 Conclusion

This study comprehensively analyzes CHSC cyclability and walkability across eastern, central, and western China. The key findings underscore the need for targeted interventions to address regional disparities and improve healthcare equity. Given China’s aging population, these disparities hold profound implications for public health planning and older adult care service delivery. Ensuring active access to primary healthcare for older adults is a critical aspect of promoting healthy aging and reducing age-related health disparities.

### 5.1 Regional disparities

Significant differences exist in the cyclability and walkability of Community Health Service Centers (CHSCs) across China’s Eastern, Central, and Western regions, with the Western region lagging. The Eastern region demonstrates the highest levels of cyclability (0.71) and walkability (0.351), reflecting its advanced urbanization, robust infrastructure, and higher density of healthcare facilities. While slightly behind in cyclability (0.64), the Central region shows better walkability (0.388) due to more effective CHSC planning and layout.

In contrast, the Western region faces significant challenges, with the lowest cyclability (0.46) and walkability (0.287) indicators. This disparity is attributed to lower urbanization levels, inadequate infrastructure, and a lower facility density (0.681 units per square kilometer). These limitations disproportionately affect older adults, who are more likely to experience physical or mobility constraints and depend on nearby healthcare services. Geographic inaccessibility can contribute to delayed care-seeking, underutilization of services, and ultimately poorer health outcomes among older adult populations.

Addressing these regional disparities requires targeted interventions, such as expanding CHSCs in the West, improving road and cycling infrastructure, and optimizing facility distribution in the East and Central regions. Such efforts not only improve spatial equity but are essential to meeting the health needs of aging populations and supporting age-friendly community development.

### 5.2 Policy recommendations

To enhance CHSC equity, promote rational medical resource distribution, and improve overall population health, this paper proposes several recommendations: increasing financial support for central and western regions to prioritize CHSC construction and optimize existing facility services; leveraging GIS technology and big data analysis to maximize facility locations and address service blind zones; enhancing urban road network density and community walking paths, particularly in the Western region, to improve walkability; strengthening the hierarchical diagnosis and treatment system by clearly distinguishing CHSCs from general hospitals; and developing a scientific evaluation system to monitor and assess CHSC service levels and equity regularly.

From an aging and public health perspective, these strategies are especially valuable for ensuring that older adult individuals receive accessible, affordable, and continuous care within their communities. This aligns with China’s Healthy Aging policy agenda and contributes to the creation of equitable, age-inclusive healthcare systems.

### 5.3 Limitations

This study has several limitations: while walkability and cyclability are critical factors for vulnerable populations such as the older adults and low-income groups, the analysis primarily focuses on walking and cycling accessibility, excluding other transportation modes like public transit, which would require additional data (e.g., transit routes, schedules) and methodological adjustments for a more comprehensive multi-modal approach. Additionally, the study is limited to 110 exemplary CHSCs across eastern, central, and western China, which, although representative, do not encompass the full diversity of the 37,177 CHSCs nationwide, necessitating future research to expand the geographic scope using high-performance computing resources for a more comprehensive understanding of CHSC accessibility and equity.

### 5.4 Future research directions

To address these limitations, future research could incorporate multiple transportation modes to evaluate accessibility more comprehensively, expand the geographic scope to include all CHSCs nationwide by leveraging high-performance computing resources for processing large-scale datasets, integrate additional data sources such as patient satisfaction surveys or environmental factors to enhance the accuracy and relevance of the analysis and develop dynamic models to capture temporal changes in accessibility and equity, thereby providing a more nuanced understanding of CHSC performance over time.

In particular, incorporating aging-related health indicators and patient usage data could better inform how CHSC spatial accessibility affects older adult care outcomes, supporting public health interventions tailored to the needs of aging populations.

### 5.5 Outlook

In conclusion, enhancing the cyclability and walkability of Community Health Service Centers (CHSCs) is essential for improving healthcare resource equity and promoting sustainable public health development in China. This is particularly important in the context of population aging, as older adults often face mobility limitations and depend heavily on nearby primary healthcare facilities for managing chronic illnesses and maintaining daily well-being. As the country faces the dual challenges of rapid urbanization and an aging population, ensuring equitable access to primary healthcare services has become a critical priority. Policymakers can address regional disparities and enhance healthcare delivery by optimizing the spatial distribution of CHSCs, improving urban infrastructure, and leveraging advanced technologies such as GIS and big data analytics.

The findings of this study highlight the urgent need to prioritize resource allocation in underserved regions, particularly in Western China, where cyclability and walkability indicators lag significantly behind those in the Eastern and Central regions. Targeted interventions, such as increasing the number of CHSCs, enhancing road network density, and promoting pedestrian-friendly urban design, can help bridge these gaps and ensure that all residents, especially vulnerable populations such as the older adults and low-income groups, have access to essential healthcare services.

Furthermore, integrating multimodal transportation planning—including walking, cycling, and public transit—can provide a more comprehensive approach to improving healthcare accessibility. Future research should explore the potential of innovative city technologies, such as real-time traffic monitoring and dynamic resource allocation, to further optimize the performance of CHSCs. Additionally, developing a robust evaluation system to regularly assess CHSC service levels and equity will be crucial for sustaining long-term improvements in healthcare accessibility. Incorporating age-specific metrics into this system can better inform public health policy and ensure that the evolving needs of older adults are addressed effectively.

Ultimately, the equitable distribution of healthcare resources is a matter of social justice and a cornerstone of sustainable development. By addressing the cyclability and walkability challenges identified in this study, China can better meet the rising demand for medical services in an aging society, strengthen community-based care for older adults, and relieve pressure on tertiary hospitals. This will contribute to achieving health equity, enhancing quality of life, and building a healthier China for future generations.

## Data Availability

The original contributions presented in the study are included in the article/supplementary material, further inquiries can be directed to the corresponding author.

## Footnotes

^1^ www.walkscore.com
