# Corrigendum: Evaluating the equity of access to community health service centers in different regions of China based on cyclability and walkability

**DOI:** 10.3389/fpubh.2025.1625081

**Published:** 2025-06-04

**Authors:** Zhenhua Xu, Jiaying Chang, Cong Han

**Affiliations:** ^1^School of Urban Construction, Beijing City University, Beijing, China; ^2^Shenzhen International Graduate School, Tsinghua University, Beijing, China; ^3^Beijing Hengchuang Institute of Additive Manufacturing Research Co., Ltd., Beijing, China; ^4^School of Architecture and Art, Hebei University of Architecture, Hebei, China; ^5^School of Architecture, University of Manchester, Manchester, United Kingdom

**Keywords:** community health service centers, cyclability, walkability, GIS analysis, equity

In the published article, there was an error. The wrong value was used for the radius.

A correction has been made to Abstract. This sentence previously stated:

“Results show that the cyclability scores for the eastern, central, and western regions were 0.71, 0.64, and 0.46, respectively, below the national standard of a 3,000-meter service radius, highlighting insufficient cycling access to primary care for older residents.”

The corrected sentence appears below:

“Results show that the cyclability scores for the eastern, central, and western regions were 0.71, 0.64, and 0.46, respectively, below the national standard of a 1,000-meter service radius, highlighting insufficient cycling access to primary care for older residents.”

The authors apologize for this error and state that this does not change the scientific conclusions of the article in any way. The original article has been updated.

